# Perceptions and intentions toward medical assistance in dying among Canadian medical students

**DOI:** 10.1186/s12910-019-0356-z

**Published:** 2019-04-02

**Authors:** James Falconer, Félix Couture, Koray K. Demir, Michael Lang, Zachary Shefman, Mark Woo

**Affiliations:** 1 Statistics Canada, Ottawa, Ontario Canada; 2 Department of Surgery, Division of Urology, Centre Hospitalier Universitaire de Sherbrooke, Sherbrooke, Québec Canada; 3 Department of Medicine, McGill University Health Centre, Montreal, Québec Canada; 4 Department of Human Genetics, McGill University, Montreal, Québec Canada; 5 Ottawa, Ontario Canada; 6 Department of Family Medicine, University of Ottawa, Ottawa, Ontario Canada

**Keywords:** Medical assistance in dying, Assisted suicide, Medical students, Canada

## Abstract

**Background:**

Medical assistance in dying (MAID) was legalized in Canada in 2016. As of July 2017, approximately 2149 patients have accessed MAID. There remains no national-level data on the perspectives of future physicians about MAID or its changing legal status. We provide evidence from a national survey of Canadian medical students about their opinions, intentions, and concerns about MAID.

**Methods:**

From October 2016 to July 2017, we distributed an anonymous online survey to all students at 15 of Canada’s 17 medical schools. The survey collected data on respondent socio-demographic characteristics, features of their medical education, intentions for medical practice, and perspectives on MAID. We analyzed responses using univariate descriptive and stepwise multivariate logistic regression.

**Results:**

In 1210 completed surveys, 71% of respondents reported being willing to provide MAID under a legal framework that permits it. Non-religious respondents reported greater willingness to participate in MAID than respondents of any religious affiliation (*p* < 0.001). Frequency of religious attendance was inversely associated with willingness to provide MAID (*p* < 0.001). Medical students born in Québec were more willing to provide MAID than respondents from other provinces (OR 2.21; *p* < 0.001). Age, sex, socioeconomic status, year of medical study, previous academic major, and rural/urban city of birth were not associated with willingness to provide MAID.

**Conclusion:**

As the current class of medical students becomes the first cohort of new physicians to enter Canada’s changing medical and legal landscape around MAID, our findings inform the public debate by examining attributes associated with support or opposition to the practice.

**Electronic supplementary material:**

The online version of this article (10.1186/s12910-019-0356-z) contains supplementary material, which is available to authorized users.

## Background

Several countries have legalized medical assistance in dying (MAID), also known as “physician-assisted dying” or “physician-assisted suicide,” over the past two decades. As of 2018, MAID was legal in Belgium, Colombia, Luxembourg, the Netherlands, Switzerland, and several U.S. states (California, Colorado, Montana, Oregon, Vermont, Washington state, and Washington D.C.), and will become legal in the state of Victoria (Australia) and Hawaii (U.S.) in 2019 [1–4]. MAID was illegal in Canada until 2016. Moreover, a 1993 Supreme Court of Canada ruling in the case of *Rodriguez v. Canada* upheld the criminal prohibition of MAID [5]. However, due to gradual changes in public opinion on the issue, public discourse and activism culminated in the landmark 2015 Supreme Court case *Carter v. Canada* [6]. In this case, plaintiffs affected by degenerative and terminal diseases successfully argued that restricting assisted suicide either inhumanely prolonged their intolerable suffering, or forced them to end their lives while still physically capable of doing so themselves, depriving them of quality years of life.

In its 2015 *Carter* decision, the Supreme Court unanimously ruled that criminal prohibitions on assisted suicide violated the right to life, liberty, and security of the person protected by Section 7 of the *Canadian Charter of Rights and Freedoms*, and the prohibition on MAID was struck down [6]*.* The Canadian government then proceeded to draft legislation regulating the practice of MAID according to the Court’s recommendations. The Canadian House of Commons introduced the “*Act to Amend the Criminal Code and to Make Related Amendments to Other Acts (Medical Assistance in Dying)*” (more commonly known as Bill C-14), in 2015, establishing the criteria for the legal provision of MAID [7]. In June 2016, the legislation was passed into law and MAID was legalized across Canada. As of June 2017, approximately 2149 patients have accessed a medically-assisted death [8].

Medical participation in end-of-life decision-making has long been a topic of ethical debate in Canada. The opinions and concerns of practicing physicians have contributed to that debate through surveys conducted in other national [9–11] and international [12–14] contexts. However, previous Canadian studies have not investigated MAID directly, but have instead focussed on related but separate issues such as palliative sedation. Since the Supreme Court’s ruling in February 2015, the opinions of Canadian medical students on the emerging practice of medical assistance in dying (MAID) have not been systematically studied. There is currently no empirical national-level data on the perspectives of future physicians about MAID or its changing legal status. Evidence-based medical practice, guidelines, and curricula will depend on reliable data for adapting policies to accommodate patient demand for this new service and physician concerns about providing it. A recent survey at McMaster University in Ontario, Canada, suggests that medical students at that school are generally open to participating in MAID, but would like training to be better incorporated within their curriculum [15]. As future physicians, medical student perspectives on this emerging practice will influence the role they will play in the provision of MAID, directly impacting patient access and quality of care across Canada. The purpose of this study is to provide the first empirical, national-level data on the opinions and intentions of Canada’s next cohort of physicians regarding MAID as they enter practice in a changed medical and legal landscape.

## Methods

We developed a survey questionnaire for distribution to all medical students at each of Canada’s medical schools. Our sampling frame included all medical students in all years of study during the 2016–2017 academic year. The invitation to participate in the study was distributed via email sent by the faculty of medicine or the medical student association at each medical school. From October 2016 to July 2017 we collected responses from an anonymous online survey distributed to medical students at 15 of Canada’s 17 medical schools. Respondents were incentivized with the option to enter a draw for one of five prizes of $100, and were informed that their draw entry could not be linked to survey responses. The study received research ethics approval from McGill University, University of Toronto, University of British Columbia, University of Alberta, University of Saskatchewan, and Memorial University of Newfoundland. The invitation to participate in research, the informed consent statement, and the survey questionnaire are included as an Additional file 1.

The survey questionnaire consisted of 47 multiple-choice and short-response questions divided into five sections measuring the respondent’s socio-demographic background (including sex, age, religion, birthplace, highest level of education reached by either parent, and educational background), experience in medical school, professional expectations and ambitions, opinions and perspectives on MAID, and their reactions to clinical scenarios involving MAID.

Our survey employed the term “physician-assisted dying” (PAD), which was the term used by the Supreme Court of Canada in their 2015 *Carter* decision. The term “medical assistance in dying” (MAID) has become preferred in recent years, including in Canadian federal and provincial legislation, to acknowledge the potential contributions of nurse practitioners and other health care professionals. Both terms are understood to include assistance from a health professional to obtain or administer medication or other treatment that intentionally brings about the patient’s own death. The questionnaire was extensively pilot-tested by approximately 50 practicing physicians, community stakeholders, activists with a range of opinions on MAID, and faculty members in medicine, law, and social science, to detect any errors or perceived bias in the wording of survey questions. The survey questionnaire was available in English and French to all respondents, and is included in Additional files 1, 2 and 3.

We used univariate descriptive analysis to measure the willingness of medical students to participate in MAID according to various socio-demographic characteristics and medical school backgrounds. We used stepwise multivariate logistic regression analyses to estimate the relative influence of several medical-student characteristics on their willingness to participate in the provision of MAID.

## Results

We received 1210 completed questionnaires. The response rate from each medical school is presented in Table 1.

**Table 1:** Participation rate by Canadian medical school

Medical school	Responses	Enrolment [31]	Participation
Dalhousie	44	456	10%
McGill	89	752	12%
McMaster	Refused	619	0%
Memorial	29	309	9%
Northern Ontario School of Medicine	50	258	19%
Queens	23	402	6%
U Alberta	133	660	20%
U Calgary	69	486	14%
U Laval	57	1116	5%
U Manitoba	94	449	21%
U Montreal	95	1402	7%
U Ottawa	127	662	19%
U Saskatchewan	82	399	21%
U Sherbrooke	Refused	819	0%
U Toronto	39	1044	4%
U British Columbia	138	1169	12%
Western	141	683	21%
Total (of sampling frame)	1210	10,247	12%
Total (of Canadian medical school enrolment)	1210	11,685	10%

**Fig. 1:**
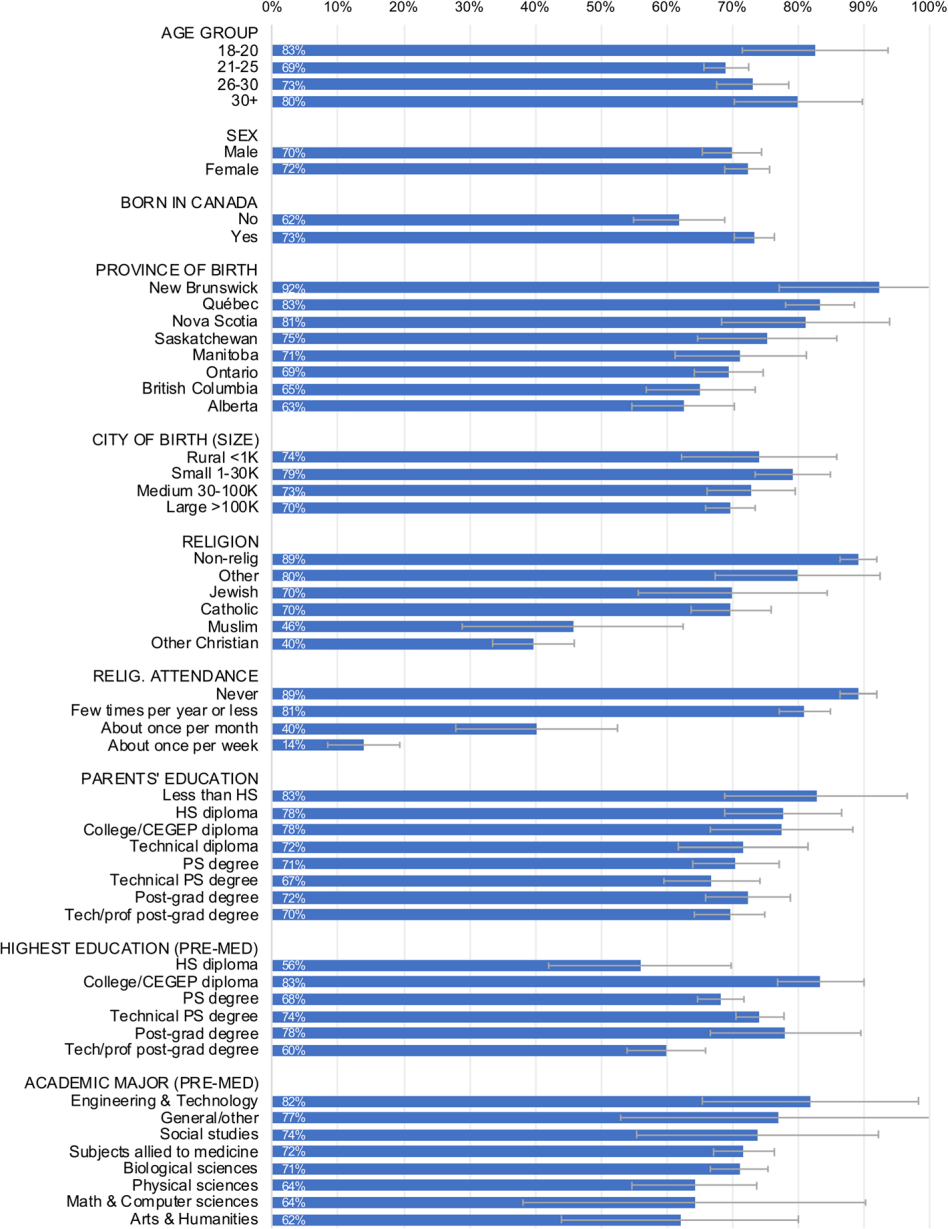
Mean willingness to participate in medical assistance in dying by socio-demographic subgroup, with 95% confidence interval

Figure 1 reports the unadjusted proportion of medical students willing to participate in MAID according to various socio-demographic characteristics, with 95% confidence intervals. Willingness to participate in MAID was measured using the question “Will you be personally willing to provide physician assisted dying (PAD), or generally participate in the process of PAD, under a legal framework that permits it?” with response options “No” or “Yes”. Respondent sub-categories with fewer than five respondents, or with proportions of 0% or 100%, were suppressed to protect the anonymity of participants. Overall, 71% of respondents expressed willingness to participate in MAID, but with significant variation across some respondent characteristics.

Respondents in the 21–25 age group, the largest age group among medical students in this study, reported the lowest willingness to participate in MAID. However, differences between the age groups were not statistically significant. Nor did we find statistically significant differences in willingness to provide MAID by sex.

Medical students born outside of Canada showed a significantly lower willingness to participate in MAID (62%) than those born or raised in Canada (73%) (*p* = 0.002). The effect remained significant when controlling for every type of religion. However, it was attenuated by controls for frequency of religious attendance.

Among medical students born in Canada, those born and/or raised in New Brunswick reported the greatest willingness to provide MAID (92%), followed by Québec (83%), Nova Scotia (81%), Saskatchewan (75%), Manitoba (71%), Ontario (69%), British Columbia (65%), and Alberta (63%). A bivariate logistic regression model showed that both the elevated rate of willingness to participate in MAID in Québec, and the reduced willingness in Alberta, were statistically distinct from the other provinces. (Québec: OR 2.20, 95% CI 1.47–3.32, *p* < 0.001; Alberta: OR 0.59, 95% CI 0.41–0.85, *p* = 0.005).

Religion and frequency of religious attendance were strongly correlated with willingness to provide MAID. Medical students who report being non-religious, atheist, or agnostic report the highest willingness to participate in MAID (89%), followed by Jewish (70%), Catholic (70%), Muslim (46%), and Other Christian religion (40%), which included all reported Christian denominations other than Catholic. A bivariate ANOVA (analysis of variance; not shown) confirmed that religious between-group variances are statistically significant at the *p* < 0.001 level. A deeper investigation revealed an interaction effect between religion and province of birth: Catholic respondents born in Québec reported a higher willingness to provide MAID (84%) compared to Catholic respondents in the rest of Canada (63%), a statistically significant difference (*p* = 0.026). No other province or religion showed an interaction effect other than that of Québec Catholics.

While type of religion strongly predicted willingness to provide MAID, so too did frequency of religious attendance. Medical students who report never attending religious services were more willing to provide MAID (89%) than those who attend a few times per year or less (81%), about once per month (40%), or about once per week (14%). The effect of religious attendance remained strongly significant (*p* < 0.001) whether modelled as a linear or categorical predictor.

Table 2 reports the results of a stepwise logistic regression model for the effect of the three most significant variables (province of birth and/or childhood, type of religion, and frequency of religious attendance) on willingness to participate in MAID. In Model 1, we see that the likelihood of participating in MAID among respondents from Québec and Alberta are significantly different from the rest of the provinces. However, the pseudo-R^2^ value of less than 3% suggests that province of origin is not a major explanatory variable for differences in willingness to participate in MAID.

**Table 2:** Willingness to participate in MAID by province of origin, religion, and frequency of religious attendance: Logistic regression odds-ratios with (standard errors)

Dependent variable: Willingness to participate in MAID, if legalized	Model 1: Province of birth	Model 2: + Religion	Model 3: +Frequency of religious attendance
Province of origin			
Alberta	0.589** (0.111)	0.715 (0.153)	0.888 (0.225)
British Columbia	0.679 (0.136)	0.594* (0.139)	0.883 (0.248)
Manitoba	0.947 (0.244)	1.097 (0.323)	1.070 (0.363)
New Brunswick	4.669 (4.871)	4.342 (4.766)	10.661 (13.546)
Nova Scotia	1.674 (0.713)	2.362 (1.091)	1.406 (0.717)
Ontario	0.821 (0.125)	0.734 (0.128)	0.773 (0.152)
Québec	2.205*** (0.459)	1.752* (0.401)	0.954 (0.231)
Saskatchewan	1.189 (0.353)	1.752 (0.602)	2.041 (0.825)
Religion			
Non-religious		7.046*** (1.286)	1.761 (0.512)
Catholic		0.730 (0.132)	1.087 (0.255)
Other Christian		0.147*** (0.026)	0.559* (0.135)
Jewish		1.015 (0.407)	1.066 (0.482)
Muslim		0.386* (0.168)	1.264 (0.754)
Other		1.554 (0.732)	1.015 (0.515)
Frequency of religious attendance (reference category: Never)			
A few times per year			0.785 (0.232)
About once per month			0.150*** (0.061)
About once per week			0.032*** (0.013)
Observations	968	963	961
Pseudo-R^2^	0.027	0.187	0.318

* P ≤ .05; ** *P* ≤ .01; *** *P* ≤ .001

Model 2 additionally controls for type of religion. Respondents who report being non-religious show an increased willingness, while Christian and Muslim respondents show a reduced willingness to participate in MAID. The effect of being born and/or raised in Québec remained statistically significant despite controlling for type of religion. The single addition of religion as a variable in our multivariate model raises the explanatory power (pseudo-R^2^) of the model to 18.7%, indicating that a major portion of the variance in willingness to participate in MAID is explained by type of religion.

Finally, Model 3 additionally controls for frequency of religious attendance. Compared to respondents who “never” attend religious services, those who attend “a few times per year” are not statistically distinct. However, respondents who attend religious services “about once per month” or “about once per week” show a strongly reduced willingness to participate in MAID (about 15%, and 3.2%, respectively), controlling for province of origin and type of religion. The reduced willingness among “Other Christians” remains significant despite controls for province of origin and frequency of religious attendance, but the effects of province of origin and all other religious categories are attenuated below the significance threshold. Moreover, the addition of frequency of religious attendance further increases the explanatory power (pseudo-R^2^) of this model to 31.8%. It appears that frequency of religious attendance, and to a lesser extent type of religion, dominate among the factors that explain willingness to participate in MAID.

Several other sociodemographic variables measured in this survey, such as urban/rural upbringing, parental education, year of study in medical school, and previous academic major before medical school, did not correlate with willingness to provide MAID.

## Discussion

As with other controversial but legal medical practices, there may be a concern that MAID will not be equitably available to patients across all regions of Canada [16]. Indeed, the preliminary findings from this study suggest a high proportion of Québec medical students willing to provide the service, and a low proportion in Alberta. However, three findings from this study should alleviate concerns about disparities in the availability of MAID across the regions of Canada. First, a majority of medical students from all provinces indicated willingness to participate in MAID, with a low of 63% in Alberta. Second, there were no statistically significant differences between medical students with an urban versus rural upbringing, potentially alleviating concerns about access to MAID outside of urban centres. Third, differences in willingness to participate in MAID are better explained by individual physician characteristics, such as type and frequency of religious attendance, which are unlikely to be systematically concentrated by geographic region.

Religion has emerged as one of the most consistently important predictors of opinion on medical assistance in dying in the international literature [17–27]. Our results show some interesting findings about the relationship between religion and willingness to provide MAID in Canada using two measures: type of religion and frequency of religious attendance. Medical students who identify as Jewish, Catholic, Muslim, or other Christian religion show significantly reduced willingness to participate in MAID relative to respondents who report being non-religious, and frequency of religious attendance showed a strong inverse association with support for MAID. However, Catholics in Québec showed significantly higher support for MAID than Catholics in the rest of Canada. The relatively high level of willingness to provide MAID among Catholic medical students was surprising considering the Catholic Church’s traditionally conservative positions on MAID and other controversial medical issues. Only controlling for the *frequency* of religious attendance eliminated the differences between Québec Catholics and those in the rest of Canada. One potential explanation is the high number of nominally-identifying Catholics in Québec who only infrequently attend Catholic religious services. If there is an association between frequency of exposure to Catholic doctrine and willingness to participate in MAID, then perhaps Québec medical students are less likely to be exposed, or to strongly adhere to it.

The greater willingness to provide MAID among medical students from Québec might be associated with the earlier public discussion and media coverage of MAID in this province, and its earlier legalization in 2014. For example, Lee et al. found that physician attitudes toward MAID were more permissive in Oregon, where it was legalized earlier than in the rest of the United States [28], and so a similar effect might be operating in Québec. Moreover, Beauvais, Mann, & Lore report that Québec, compared to the United States and other Canadian provinces, exhibits more liberal attitudes toward controversial medical practices such as abortion, which could also partially explain this significant difference in Québec [29].

Immigrant status was associated with reduced support for MAID, but this association was explained away by controlling for frequency of religious attendance. The correlation between being born in Canada and support for MAID is therefore better explained by the higher proportion of foreign-born medical students who report being religious. Other variables, such as age, did not significantly correlate with willingness to provide MAID in our sample. Previous evidence about the effect of age on opinion about MAID is mixed [26, 30], but our results conform to findings from Lucchetti et al. who reported no effect of age [23].

## Limitations

With over 1200 respondents, this is, to our knowledge, the largest study of medical student attitudes toward MAID conducted in North America. Nevertheless, there is a risk of a non-random self-selection bias among respondents who participated, or declined to participate, in the survey. Two of Canada’s 17 medical schools refused to allow their students to participate in the survey: Université de Sherbrooke (Québec), and McMaster University (Ontario), which was conducting their own internal survey of student attitudes around the same time [15]. The non-participation of these medical schools may affect the national representativeness of this survey. However, our survey achieved coverage of other universities in Ontario and Québec, ensuring that those provinces were still adequately represented in the data. Our descriptive results should therefore not be considered generalizable to the overall population of medical students in Canada. Rather, our main findings are based on the relationships between medical student attributes and their opinions and perspectives of MAID, which can be validly inferred from these analyses.

## Conclusion

Amid much public debate, MAID was legalized in Canada in 2016. However, there remains a dearth of national-level empirical evidence on the opinions and intentions of doctors and medical students about their willingness to provide this new type of practice. As the next cohort of Canadian physicians prepares to enter medical practice under a changing legal landscape, more research is needed to investigate whether Bill C-14 adequately responds to the needs and concerns of patients and physicians in the clinical setting.

We provide a systematic measurement of Canadian medical student opinion by surveying students at 15 of Canada’s 17 medical schools. Medical students from Québec and those who report being non-religious were significantly more willing to participate in MAID. Medical students who identify as Christian, and medical students of any religion who attend religious services more frequently than once per month, were significantly less likely to participate in MAID. Type of religion and frequency of religious attendance emerged as dominant variables, overshadowing the effect of province of birth, or of being born outside Canada. Our findings provide the first national-level data of medical student opinion on the legalization of MAID since this monumental change in Canada’s medical and legal landscape, and will inform the ongoing public debate around this emerging medical practice.

## Additional files

Additional file 1: Survey questionnaire: Perceptions of physician assisted dying among Canadian medical students: implications for policy and practice. (PDF 545 kb)

Additional file 2: Invitation to Research. The text of the first email contact with survey participants inviting them to participate in the study. (DOCX 121 kb)

Additional file 3: Informed Consent document. Copy of the informed consent document that preceded the online survey questionnaire. (DOCX 142 kb)

## Abbreviations

ANOVA: Analysis of variance
CI: Confidence Interval (95%)
MAID: Medical assistance in dying
OR: Odds-Ratio
PAD: Physician-assisted dying
v.: Versus
