# Supplementary material for: Perceptions and intentions toward medical assistance in dying among Canadian medical students
Source: BMC Med Ethics. 2019 Apr 2;20:22. doi: 10.1186/s12910-019-0356-z (PMC6444527; doi:10.1186/s12910-019-0356-z)
Supplement: Supplementary file 1 — Survey questionnaire: Perceptions of physician assisted dying among Canadian medical students: implications for policy and practice. (PDF 545 kb) [file 12910_2019_356_MOESM1_ESM.pdf]

Perceptions of physician-assisted dying among Canadian medical students: implications for policy and practice

This survey will capture meaningful responses from people across the spectrum of opinions about physician-assisted dying (PAD), whether they oppose, support, or have no opinion about PAD. If you feel you have been unable to adequately express your position about PAD, please indicate so in the comment boxes following each section of the survey. We hope we have written these survey questions relatively free from bias. However, if you object to the wording of any question, or if you detect bias in any question or its response options, please comment in the textbox associated with each question. We will never publish survey responses that could identify you. Even if you feel that your combination of characteristics makes you uniquely identifiable, we will never publish results from a single respondent. Your responses will be combined with others' to publish overall rates and trends. Following the survey, you will be redirected to an external website where you can choose to enter a draw for one of 5 cash prizes of $100. Your name and email address collected for the prize draw are not and cannot be linked to your survey responses - not even using survey metadata (i.e., we don't collect your internet IP address, or time-stamp your submission). Winners will be personally notified via email immediately after the survey is closed on 30 Nov 2016. Obviously, due to anonymity, we cannot publicize the identity of winners.
You are invited to participate in a study of Canadian medical students, to survey your views and perceptions on physician-assisted dying. As future medical practitioners, your opinions on this issue are extremely valuable. Students with any range of opinions (or even no opinion) on physician-assisted dying are encouraged to contribute to this study. Your status as a current medical student in a Canadian university has made you eligible for this study. All current medical students in Canada will receive an invitation to this survey. Participation is voluntary.

Purpose: No empirical data currently exist on the perceptions of medical students about the issue of physician-assisted dying (also called medical assistance in dying) in Canada. An evidence basis is needed for the creation of policy, legal frameworks, professional guidelines, and medical education. Your participation will significantly contribute to the success of this study.

Requirements: Participation in this study requires you to complete a 15-minute anonymous survey. You will be asked questions about your views, opinions, perceptions, and experiences about physician-assisted dying, as well as some questions about your background, future ambitions, and medical education. Please pay close attention to the questions and response options, and answer honestly.

Privacy: This survey is completely anonymous and does not collect any personally-identifying information or metadata. Results will not be published in a way that could identify respondents.

Risks: Due to the subject matter of the survey, there is a risk of psychological or emotional discomfort. You may refuse to answer any question or withdraw from the study at any time. Because the survey is anonymous, we cannot identify your responses to withdraw you from the study after you have completed and submitted your survey). If you experience distress at any time during the completion of this survey and require assistance, please contact the counselling and mental health service at your university at **this link**.

There is a risk of personal, social, professional, or legal consequences if you disclose any illegal or unethical activity during the survey. We do not ask about illegal or unethical activity, and you should not disclose it during the survey. We guarantee anonymity for your survey responses to protect against these risks. We have designed the data collection such that no researcher can identify you, even if we

wanted to. You should complete the survey privately and keep your responses private. Survey data will be stored for an indefinite period on an encrypted, password-protected hard drive and made accessible exclusively to analysts on the project team.

Benefits: There is no personal benefit to participating in this study. However, this study has benefits for the profession of medicine and medical education. No Canadian data currently exist on the perspective of medical students on the issue of physician-assisted dying. This research will provide important data on how future physicians perceive this practice on an ethical, social, and professional level. Survey results will be useful for drawing evidence-based conclusions on the future of physician-assisted dying in Canada, and for recommendations on regulatory frameworks, professional guidelines, and medical education. We will publish the results of this research through reports, academic presentations, policy papers, and journal articles.

Compensation: There is no compensation for participating in this study. However, following the survey, you will be redirected to an external webpage where you can choose to enter a draw to win one of 5 cash prizes of $100. Entering the draw is voluntary. Your name and email collected for the purposes of the prize draw cannot be linked to your survey responses in any way, will only be accessible to one independent analyst on the research team (Zachary Shefman), and will be securely deleted following the draw.

Conflict of interest: The researchers have no personal, professional, or financial stake in the results of this survey or the legal status of medical assistance in dying. We are an independent and non-partisan research team, not sponsored by any public or private stakeholders, institutions, or corporations, and with no conflicts of interest.

Thank you.

This study has been reviewed and given ethics approval by the *McGill Research Ethics Board* and the *Newfoundland and Labrador Health Research Ethics Board*. If you have any questions about taking part in this study, you can contact the principal investigator, James Falconer, at mcgillmedpass@gmail.com. Or you can talk to someone who is not involved with the study, but who can advise you on your rights as a participant in a research study, by contacting the McGill Ethics Manager at 514-398-6831, or lynda.mcneil@mcgill.ca. In Newfoundland and Labrador, you can contact the Ethics Office at 709-777-6974, or email info@hrea.ca. [Click here to download or print a copy of this consent form for your records]

Researchers:
Dr. James Falconer (Principal Investigator), Dept. of Sociology, University of Alberta
Félix Couture, MDCM candidate, Faculty of Medicine*
Koray Demir, MDCM candidate, Faculty of Medicine*
Michael Lang, BCL/LLB candidate, Faculty of Law*
Zachary Shefman, BCL/LLB candidate, Faculty of Law*
Mark Woo, MDCM candidate, Faculty of Medicine*
*McGill University, Montreal, Quebec, Canada
Project email: mcgillmedpass@gmail.com

Supervisor: Dr. Jennifer Fishman, McGill University
Montreal, Quebec, Canada
jennifer.fishman@mcgill.ca
514-398-7403

There are 52 questions in this survey

Socio-demographic

1

Socio-Demographic Section

The following questions collect basic social and demographic information about you

2

Please enter your age in years.

Please write your answer here:

[ ]

3

Please select your sex or gender.

Please choose **only one** of the following:

- ○ Female
- ○ Male
- ○ Prefer not to answer
- ○ Other [ ]

4

Were you born in Canada?

Please choose **only one** of the following:

- ○ Yes
- ○ No

5

Please select the Canadian province or territory in which you were born.

Only answer this question if the following conditions are met:
° ((**SD03.NAOK** != "N"))

Please choose **only one** of the following:

- Alberta
- British Columbia
- Manitoba
- New Brunswick
- Newfoundland and Labrador
- Northwest Territories
- Nova Scotia
- Nunavut
- Ontario
- Prince Edward Island
- Quebec
- Saskatchewan
- Yukon

6

If you were not born in Canada, did you live in Canada for the majority of your childhood (up to age 18)?

Only answer this question if the following conditions are met:
° ((**SD03.NAOK** != "Y"))

Please choose **only one** of the following:

- Yes
- No

7

Please select the Canadian province or territory where you lived for the majority of your childhood (up to age 18).

Only answer this question if the following conditions are met:
° ((**SD03.2.NAOK** != "N"))

Please choose **only one** of the following:

- Alberta
- British Columbia
- Manitoba
- New Brunswick
- Newfoundland and Labrador
- Northwest Territories
- Nova Scotia
- Nunavut
- Ontario
- Prince Edward Island
- Quebec
- Saskatchewan
- Yukon

8

What population size was the Canadian city or town in which you were born and/or lived most of your childhood?

Only answer this question if the following conditions are met:
° ((**SD03.NAOK** != "N")) or ((**SD03.NAOK** != "Y") and (**SD03.2.NAOK** != "N"))

Please choose **only one** of the following:

- Rural area (population of less than 1,000 people)
- Small population centre (population 1,000 to 29,999)
- Medium population centre (population 30,000 to 99,999)
- Large urban population centre (population 100,000 or more)

9

How often do you attend religious services?

Please choose **only one** of the following:

- ○ Never
- ○ A few times per year or less
- ○ About once per month
- ○ About once per week
- ○ About once per day or more

10

What is your religion, or what type of religious services do you attend?

Please choose **all** that apply:

- ☐ Non-religious, atheist, agnostic
- ☐ Buddhist
- ☐ Chinese traditional
- ☐ Christian: Catholic
- ☐ Christian: Protestant
- ☐ Christian: Other (specify in comments)
- ☐ Hindu
- ☐ Indigenous/aboriginal religion
- ☐ Jewish
- ☐ Muslim
- ☐ Other Eastern religion (specify in comments)
- ☐ Other: ________

11

What was the highest level of schooling achieved by your parent or caregiver who was your household's primary income earner when you were a child (up to age 18)?

Please choose **only one** of the following:

- Less than high school
- High school diploma
- College/CEGEP diploma or certificate
- Technical diploma or certificate (e.g., skilled trade, professional accreditation)
- Post-secondary degree (e.g., Bachelor's, Baccalaureate)
- Post-secondary degree in a technical/professional field (e.g., engineering, nursing, business)
- Post-graduate degree (e.g., Master's, PhD)
- Post-graduate degree in a technical/professional field (e.g., law, medicine, accounting, MBA)
- Don't know
- Other

12

What was the highest level of schooling achieved by your parent or caregiver who was your household's secondary income earner (if applicable) when you were a child (up to age 18)?

Please choose **only one** of the following:

- Not applicable / no second parent (single-parent family)
- Less than high school
- High school diploma
- College/CEGEP diploma or certificate
- Technical diploma or certificate (e.g., skilled trade, professional accreditation)
- Post-secondary degree (e.g., Bachelor's, Baccalaureate)
- Post-secondary degree in a technical/professional field (e.g., engineering, nursing, business)
- Post-graduate degree (e.g., Master's, PhD)
- Post-graduate degree in a technical/professional field (e.g., law, medicine, accounting, MBA)
- Don't know
- Other

13

What was your highest level of schooling completed before medical school?

Please choose **only one** of the following:

- Less than high school
- High school diploma
- College/CEGEP diploma or certificate
- Technical diploma or certificate (e.g., skilled trade, professional accreditation)
- Post-secondary degree (e.g., Bachelor's, Baccalaureate)
- Post-secondary degree in technical/professional field (e.g., engineering, nursing, business)
- Post-graduate degree (e.g., Master's, PhD)
- Post-graduate degree in technical/professional field (e.g., law, medicine, accounting, MBA)
- Other ____________

14

What was your academic major and minor (if applicable) before medical school?

Please write your answer(s) here:

Major: ____________

Minor (if applicable): ____________

Medical School

15

Medical School Section

The following questions collect information about your medical education and experience

16

Which Canadian medical school do you attend?

Please choose **only one** of the following:

- ◯ Dalhousie University
- ◯ McGill University
- ◯ McMaster University (Michael G. DeGroote School of Medicine)
- ◯ Memorial University of Newfoundland
- ◯ Northern Ontario School of Medicine
- ◯ Queen's University
- ◯ University of Alberta
- ◯ University of British Columbia
- ◯ University of Calgary (Cumming School of Medicine)
- ◯ University of Manitoba
- ◯ University of Ottawa
- ◯ University of Saskatchewan
- ◯ University of Toronto
- ◯ Université de Montréal
- ◯ Université de Sherbrooke
- ◯ Université Laval
- ◯ Western University (Schulich School of Medicine & Dentistry)
- ◯ Other ____________

17

What is your current year of study in medical school?

Please choose **only one** of the following:

- 1st year
- 2nd year
- 3rd year
- 4th year
- 5th year or more

18

Before medical school, did you have previous clinical experience working or volunteering in any of the following?

Please choose **all** that apply:

- A health profession (e.g., nursing, physiotherapy, nutrition, etc.)
- A hospital
- A medical clinic
- None of the above
- Other:

19

Please select your current stage of medical training.

Please choose **only one** of the following:

- Pre-clinical training
- Junior clerkship
- Senior clerkship
- Other

20

Does your medical education include courses or content related to physician-assisted dying?

Please choose **only one** of the following:

- ◯ No
- ◯ Yes
- ◯ Don't know / Have not yet begun courses

21

To what extent do you think your medical school should incorporate training about physician-assisted dying (PAD) into the curriculum?

Please choose **only one** of the following:

- ◯ Medical school should **not** include training on PAD
- ◯ Medical school should expose students to some issues related to PAD
- ◯ Medical school should provide substantive training on PAD
- ◯ Other [ ]

Professional outlook

22

Professional Outlook Section

The following questions ask about your intentions for your practice of medicine after the completion of your medical residency and training

23

Do you intend to practice medicine after medical school?

Please choose **only one** of the following:

- ○ No
- ○ Yes
- ○ Unsure / undecided

24

Do you intend to remain in Canada to practice medicine?

Only answer this question if the following conditions are met:
° (((**is_empty**(**PO_01.NAOK**) || (**PO_01.NAOK** != "0"))))

Please choose **only one** of the following:

- ○ No
- ○ Yes
- ○ Unsure / undecided

25

In which Canadian province(s) or territory(ies) do you intend to practice medicine?

Only answer this question if the following conditions are met:
° (((is_empty(PO_01.NAOK) || (PO_01.NAOK != "0"))) and ((is_empty(PO_02.NAOK) || (PO_02.NAOK != "0"))))

Please choose **all** that apply:

- ☐ Alberta
- ☐ British Columbia
- ☐ Manitoba
- ☐ New Brunswick
- ☐ Newfoundland and Labrador
- ☐ Northwest Territories
- ☐ Nova Scotia
- ☐ Nunavut
- ☐ Ontario
- ☐ Prince Edward Island
- ☐ Quebec
- ☐ Saskatchewan
- ☐ Yukon

26

Please indicate the size and, if applicable, the name of the Canadian city(ies) in which you intend to practice medicine.

Only answer this question if the following conditions are met:
° (((is_empty(PO_01.NAOK) || (PO_01.NAOK != "0"))) and ((is_empty(PO_02.NAOK) || (PO_02.NAOK != "0"))))

Please choose **all** that apply and provide a comment:

☐ Rural area (population of less than 1,000 people)	
☐ Small population centre (population 1,000 to 29,999)	
☐ Medium population centre (population 30,000 to 99,999)	
☐ Large urban population centre (population 100,000 or more)	

27

What is/are your intended field(s) of medical practice or specialization(s), or to which residency specialization(s) do you intend to apply?

Only answer this question if the following conditions are met:
° (((is_empty(PO_01.NAOK) || (PO_01.NAOK != "0"))))

Please choose **all** that apply:

- ☐ Anatomical Pathology
- ☐ Anesthesiology
- ☐ Cardiac Surgery
- ☐ Dermatology
- ☐ Diagnostic Radiology
- ☐ Emergency Medicine
- ☐ Family Medicine
- ☐ General Pathology
- ☐ General Surgery
- ☐ Hematological Pathology
- ☐ Internal Medicine
- ☐ Laboratory Medicine
- ☐ Medical Biochemistry
- ☐ Medical Genetics
- ☐ Medical Microbiology
- ☐ Neurology
- ☐ Neurology - Pediatric
- ☐ Neuropathology
- ☐ Neurosurgery
- ☐ Nuclear Medicine
- ☐ Obstetrics & Gynecology
- ☐ Ophthalmology
- ☐ Orthopedic Surgery
- ☐ Otolaryngology - Head & Neck Surgery
- ☐ Pediatrics
- ☐ Physical Medicine & Rehabilitation
- ☐ Plastic Surgery
- ☐ Psychiatry
- ☐ Public Health & Preventive Medicine
- ☐ Radiation Oncology
- ☐ Urology
- ☐ Vascular Surgery

☐ Other: ____________

28

How likely are you to practice in the following fields during your medical career?

Only answer this question if the following conditions are met:
° (((**is_empty**(**PO_01.NAOK**) || (**PO_01.NAOK** != "0"))))

Please choose the appropriate response for each item:

	Very unlikely	Unlikely	Likely	Very likely
Palliative Care	○	○	○	○
Oncology	○	○	○	○
Geriatrics	○	○	○	○

Topic Questions

29

Topic Questions Section

The following questions measure your opinions and experiences about Physician-Assisted Dying (PAD)

30

Have you ever observed or attended a death in any of the following settings?

Please choose **all** that apply:

- ☐ No, I have not observed or attended a death
- ☐ A patient or someone under your care
- ☐ A friend, family member, or loved one
- ☐ Other: ____________

31

Was the death of your patient, or the person under your care, preceded by a period of severe and irremediable suffering?

Only answer this question if the following conditions are met:
° ((**TQ01_1.NAOK** == "Y"))

Please choose **only one** of the following:

- ○ No
- ○ Yes
- ○ Unsure

32

Was the death of your friend, family member, or loved one preceded by a period of severe and irremediable suffering?

Only answer this question if the following conditions are met:
° ((**TQ01_2.NAOK** == "Y"))

Please choose **only one** of the following:

- ◯ No
- ◯ Yes
- ◯ Unsure

33

What is your current level of knowledge and understanding about the legal requirements for physicians to provide physician-assisted dying (PAD)?

Please choose **only one** of the following:

- ◯ I have **no knowledge** of what is required of physicians to legally provide PAD
- ◯ I have a **vague** understanding of what is required of physicians to legally provide PAD
- ◯ I have a **fairly reasonable** understanding of what is required of physicians to legally provide PAD
- ◯ I have a **complete** and detailed understanding of what is required of physicians to legally provide PAD

34

Are you aware of the Supreme Court of Canada ruling in the case of *Carter v. Canada*, which struck down Canada's prohibition on physician-assisted dying for people who meet certain criteria?

Please choose **only one** of the following:

- ◯ No
- ◯ Yes, I have heard of the Carter ruling, but I am **not** familiar with its content
- ◯ Yes, I am familiar with the Carter ruling **and** its content

35

Are you aware of the recent federal legislation responding to the Supreme Court of Canada's *Carter* decision (first introduced as Bill C-14)?

Please choose **only one** of the following:

- ○ No
- ○ Yes, I have heard of this legislation, but I am **not** familiar with its content
- ○ Yes, I am familiar with this legislation **and** its content

36

Please read carefully: Regardless of whether you support or oppose physician-assisted dying (PAD), which of the following reasons or arguments for allowing PAD do you find legitimate?

Please choose **all** that apply:

- ☐ Allowing PAD respects the autonomy of patients to make decisions about their own health, life, and death
- ☐ In some cases, PAD reduces suffering, which is compatible with the profession of medicine and the role of doctors and nurses
- ☐ PAD could conserve limited healthcare system resources
- ☐ PAD permits one to die without the stigma of suicide
- ☐ Legalizing PAD protects doctors from prosecution for a medically recognized practice
- ☐ PAD is socially acceptable to a majority of Canadians
- ☐ If PAD is legalized, then it can be regulated
- ☐ A criminal prohibition on PAD forces some people to endure intolerable suffering
- ☐ Vulnerable patients can be better protected from unwanted PAD if legal safeguards are properly designed
- ☐ There is no ethical difference between PAD and the legal practice of palliative sedation (sedation to the point of complete unconciousness with no intent to reawake the patient for the remaining duration of their life)
- ☐ There is no ethical difference between an action and an omission: Doctors already legally withhold life-sustaining treatment (omission), so they should be legally permitted to cause death (action)
- ☐ Suicide is not illegal, so it is inconsistent to permit suicide, and yet prohibit PAD
- ☐ Other: ______________________

37

Please read carefully: Regardless of whether you support or oppose physician-assisted dying (PAD), which of the following reasons or arguments for prohibiting PAD do you find legitimate?

Please choose **all** that apply:

- ☐ Life is sacred and should therefore be preserved, whatever the circumstances
- ☐ The role of medicine should be to preserve life, whatever the circumstances
- ☐ Palliative care is adequate to provide patients with relief from intolerable suffering
- ☐ No safeguards can adequately protect vulnerable persons from unwanted or coerced PAD
- ☐ No safeguards can adequately protect vulnerable persons from medical error in assessing patient consent for PAD
- ☐ The prohibition of PAD protects vulnerable persons from ending their life in a time of temporary weakness
- ☐ Participating in PAD is a violation of medical ethics
- ☐ Legalization of PAD devalues the lives of people with disabilities
- ☐ Legalization of PAD is likely to lead to legalization of euthanasia for minors, persons with psychiatric disorders, minor medical conditions, or other circumstances where it would be inappropriate
- ☐ Permitting PAD allows the state to determine, as a matter of law, which lives are worth living; we should not give the state that power
- ☐ Legalization of PAD impedes the development of palliative care
- ☐ Legalization of PAD changes the physician-patient relationship for the worse
- ☐ The informed consent standard cannot be applied in the context of PAD
- ☐ Physician bias undermines the assessment of whether the elderly, disabled, or other vulnerable patients meet the eiligibility criteria for PAD
- ☐ Other: ____________________

38

Regardless of the legal status of physician-assisted dying (PAD), medical colleges (the organizations responsible for the professional oversight of physicians) should ...

Please choose **only one** of the following:

- ○ **Not** permit their physician members to provide PAD under any circumstances
- ○ Determine acceptable medical criteria for PAD, in addition to any legal criteria, and enforce compliance by physician members
- ○ Permit its individual physician members to determine the acceptable criteria for PAD, and support the autonomy of individual physicians in their decisions
- ○ Other [ ]

39

Whose opinion on physician-assisted dying (PAD) should have more influence: medical opinion or legal opinion?

Please choose **only one** of the following:

- ○ **Legal** opinion should have more influence; physicians and medical associations should conform to what lawmakers decide on the issue
- ○ **Medical** opinion should have more influence; lawmakers and the public sector should conform to what physicians and medical associations decide on the issue

40

The Supreme Court of Canada ruling in *Carter v. Canada* permits physician-assisted dying in cases of "*grievous and irremediable medical conditions*" that cause "*enduring and intolerable suffering*" and where patients have "*the capacity to consent*". Do you feel that physicians, in general, have the expertise to assess whether a patient:

Please choose the appropriate response for each item:

	Yes	No
Has a "grievous and irremediable medical condition"?	○	○
Experiences "enduring and intolerable suffering"?	○	○
Has "the capacity to consent"?	○	○

41

The Supreme Court of Canada ruling in *Carter v. Canada* permits physician-assisted dying in cases of "*grievous and irremediable medical conditions*" that cause "*enduring and intolerable suffering*" and where patients have "*the capacity to consent*". Do you feel that now, or following the completion of your medical training, you personally will have the expertise to assess whether a patient:

Please choose the appropriate response for each item:

	Yes	No
Has a "grievous and irremediable medical condition"?	○	○
Experiences "enduring and intolerable suffering"?	○	○
Has "the capacity to consent"?	○	○

42

What do you personally think should be the legal status of physician-assisted dying (PAD)?

Please choose **only one** of the following:

- ○ PAD should remain a crime, and **all** instances should be prosecuted
- ○ PAD should remain a crime, but **not all** instances should be prosecuted
- ○ PAD should be decriminalized or legalized, with acceptable criteria determined by **legal** guidelines.
- ○ PAD should be decriminalized or legalized, with acceptable criteria determined by **medical** guidelines, without regulation from legal guidelines.
- ○ Other ____________

Clinical Scenarios

43

Clinical Scenarios Section

The following questions measure your opinions about physician-assisted dying (PAD) based on scenarios involving patients and physicians

44

For each of the following clinical scenarios, indicate whether you personally believe a patient should be permitted to access physician-assisted dying (PAD):

Please choose the appropriate response for each item:

	No	Yes
Competent patient with a **degenerative neurological condition** (e.g., Alzheimer's, Dementia, Huntington's, Parkinson's, ALS)	○	○
Patient with an irremediable medical condition that causes enduring and intolerable suffering, but whose death is **not reasonably foreseeable**	○	○
Patient with a traumatic and life-altering **physical injury** from which they will not recover (e.g., paraplegia, quadriplegia)	○	○
Mature and competent patient **below the age of 18** with a grievous and irremediable medical condition that causes enduring and intolerable suffering	○	○
Patient with grievous and intolerable **psychological suffering** (e.g., depression, anxiety, post-traumatic stress disorder)	○	○
Patient who finds all **viable treatment options unacceptable** (e.g., refuses treatment, chemotherapy, adaptations)	○	○
Patient who is currently unable to consent to PAD, but who **provided an advance directive** requesting PAD under the circumstances	○	○
Patient who is currently unable to consent to PAD, and who did **not** provide an advance directive requesting PAD, but whose **immediate family** indicate that the **patient would want PAD** under the circumstances	○	○
Patient with a grievous and irremediable medical condition that causes enduring and intolerable suffering, who **travels to Canada to receive PAD**.	○	○

45

The law requires patients to meet several criteria to be eligible for physician-assisted dying (PAD).

For each of the following patient criteria, please indicate whether you personally believe the criteria is too permissive, appropriate, or too restrictive for accessing PAD.

(Note: criteria that are too permissive would allow patients to access PAD who should not be able to; criteria that are too restrictive would prevent patients from accessing PAD who should be able to)

Please choose the appropriate response for each item:

	Too **permissive**	Appropriate	Too **restrictive**
The patient must have a **serious and incurable illness**, disease, or disability	○	○	○
The patient must be in an **advanced state of irreversible decline** in capability	○	○	○
The patient's medical condition must cause them **enduring physical or psychological suffering** that is **intolerable** to them	○	○	○
The patient's suffering **cannot be relieved** under conditions that they consider acceptable	○	○	○
The patient's natural **death has become reasonably foreseeable** (but remaining length of life need not be specified)	○	○	○
The patient must be **eligible** (or will be eligible after a waiting period) to r**eceive publicly-funded healthcare** in Canada	○	○	○
The patient must be at least **18 years of age**	○	○	○
The patient must be **capable of making decisions** about their health	○	○	○
The patient's **voluntary request** for medical assistance in dying must be made **without external pressure**	○	○	○
The patient must give **informed consent** to receive medical assistance in dying	○	○	○

46

The law requires physicians to meet several criteria to legally provide a physician-assisted death (PAD).

For each of the following physician criteria, please indicate whether you personally believe the criteria is too permissive, appropriate, or too restrictive for providing PAD.

(Note: criteria that are too permissive would allow patients to access PAD who should not be able to; criteria that are too restrictive would prevent patients from accessing PAD who should be able to)

Please choose the appropriate response for each item:

	Too **permissive**	Appropriate	Too **restrictive**
Physician must be of the opinion that the **patient meets all of the legal criteria** for eligibility	○	○	○
Physician must ensure that the patient's **request for PAD was made in writing**, and signed and dated by the patient or someone on their behalf	○	○	○
Physician must ensure that the patient's request for PAD was made **after the patient was informed** by a medical practitioner that **the patient's natural death has become reasonably foreseeable**	○	○	○
Physician must be satisfied that the request for PAD was signed and dated by the patient (or someone on their behalf) before **two independent witnesses**, who also sign and date the request	○	○	○
Physician must ensure that the patient has been informed that they **may withdraw their request for PAD at any time**, for any reason	○	○	○
Physician must ensure that a **second medical practitioner** has provided a **written opinion** confirming that the patient meets all of the criteria for PAD	○	○	○
Physician must be satisfied that they and the **other medical practitioner** providing the written opinion **are independent and have no conflict of interest**	○	○	○
Immediately before providing the medical assistance in dying, the physician must give the patient an **opportunity to withdraw their request**, and ensure that the patient gives **express consent** to receive medical assistance in dying	○	○	○
Physician must ensure that there are at least **15 days between the request for PAD and the day on which the medical assistance in dying is provided** (or a shorter period if both medical practitioners believe that the patient will imminently lose their capacity to provide express consent)	○	○	○

47

Thinking of a physician who is opposed to physician-assisted dying (PAD) but receives a request for PAD from a patient, please indicate whether you think each of the following physician reactions are appropriate or not appropriate:

Please choose the appropriate response for each item:

	**Not** appropriate	Appropriate
Refuses to provide PAD, and **obstructs the patient** from accessing PAD (e.g., by providing misinformation, delaying access)	○	○
Refuses to provide PAD, and **refuses to provide information** the patient could use to access PAD	○	○
Refuses to provide PAD, and **refuses to provide a referral** to a different physician who would provide PAD	○	○
Refuses to provide PAD by interpreting the eligibility criteria **very restrictively**	○	○
Refuses to **receive or acknowledge the patient's request** for PAD (e.g., by ignoring the request)	○	○
Refuses to provide PAD, but instead **refers the patient to counselling**	○	○
Refuses to provide PAD, but **refers** the patient to a **different physician who would provide PAD**	○	○
Refuses to provide PAD, but **assists the patient in accessing PAD**	○	○
**Provides** a PAD, **against their ethical judgement**	○	○

48

Thinking of a physician who is opposed to physician-assisted dying (PAD) but receives a request for PAD from a patient, what do you think the physician should be *required* to do by law or by medical professional regulations?

Physicians opposed to PAD ...

Please choose **all** that apply:

- ☐ Should be under **no obligation whatsoever** to provide any information, referral, assistance, or acknowledgement when faced with a patient request for PAD
- ☐ Should be obliged to **provide correct information**, but **not** a referral to a different physician who can provide PAD
- ☐ Should be obliged to **provide a referral** to a physician who can provide PAD
- ☐ Should be obliged to provide an **honest assessment of the patient's condition**, to potentially allow them to be eligible for PAD
- ☐ Should be obliged to provide either the first or second **medical opinion** necessary to be eligible for PAD
- ☐ Should be obliged to **prescribe the required dose of life-ending medication** for the patient to administer to themselves at a time of their choosing
- ☐ Should be obliged to **attend their patient's PAD**
- ☐ Should be obliged to **administer the life-ending medication** to their patient
- ☐ Other: ________

49

Please indicate whether you would be personally willing to fulfil each of the following services in the course of physician-assisted dying (PAD):

Please choose the appropriate response for each item:

	**Not** willing	Willing
Provide **honest information** to a patient inquiring about PAD	○	○
Provide a **referral to another doctor** willing to assist the patient with their request for PAD	○	○
Provide the **sole decision** approving a PAD	○	○
Provide the **first (of two) physician approvals** required for PAD	○	○
Provide the **second physician approval** for PAD, subject to prior approval by a first physician	○	○
**Prescribe a lethal dose of medication** for the patient to self-administer	○	○
**Perform the medical act** that causes death (lethal injection or other)	○	○

50

Will you be personally willing to provide physician-assisted dying (PAD), or generally participate in the process of PAD, under a legal framework that permits it?

Please choose **only one** of the following:

- ○ Yes
- ○ No

51

If you are personally willing to provide physician-assisted dying (PAD), what factors might deter or prevent you from providing PAD, or from participating in a patient's access to PAD?

Only answer this question if the following conditions are met:
° ((**CS07.NAOK** != "N"))

Please choose **all** that apply:

- ☐ Fear of **criminal prosecution** if the legal requirements are not sufficiently well-defined (i.e., being charged with a crime, criminal record, jail sentence)
- ☐ Fear of **civil legal action** if the legal requirements are not sufficiently well-defined (i.e., being sued for wrongful death, malpractice, paying damages)
- ☐ Fear of **professional consequences** (i.e., revoked medical license)
- ☐ Fear of **harassment, threats, or violence** from members of the public who oppose PAD
- ☐ Fear of **alienating patients** if they discover you are willing to provide PAD
- ☐ Fear of **professional stigma** or pressure from colleagues
- ☐ Fear of **making an error**, or uncertainty about your own capacity to assess whether a patient qualifies for PAD
- ☐ Other: ____________________

52

If you are not willing to provide physician-assisted dying (PAD), what factors motivate your decision?

Only answer this question if the following conditions are met:
° ((**CS07.NAOK** != "Y"))

Please choose **all** that apply:

- ☐ Religious reasons
- ☐ Reasons of medical ethics or professional oaths
- ☐ Personal moral/ethical objection to PAD
- ☐ Personal discomfort or unease with providing PAD
- ☐ Lack of personal interest in providing PAD
- ☐ Fear of making an error, or uncertainty about your own capacity to assess whether a patient qualifies for PAD
- ☐ Inability to ensure my own independence, or the independence of supporting medical practitioners, to evaluate PAD requests
- ☐ Fear of alienating patients by being a provider of PAD
- ☐ Fear of professional stigma
- ☐ Fear of harassment, threats, or violence from members of the public who oppose PAD
- ☐ Fear of civil legal action (i.e., being sued for wrongful death, malpractice, paying damages)
- ☐ Fear of criminal prosecution (i.e., being charged with a crime, criminal record, jail sentence)
- ☐ Fear of professional consequences (i.e., revoked medical license)
- ☐ Other: ____________

Thank you for your participation. Your answers have been saved. Please click below to be redirected to an external website where you can choose to enter a draw for one of 5 prizes of $100.
1969.12.31 – 19:00

Submit your survey.
Thank you for completing this survey.
