# Supplementary material for: Perceptions and intentions toward medical assistance in dying among Canadian medical students
Source: BMC Med Ethics. 2019 Apr 2;20:22. doi: 10.1186/s12910-019-0356-z (PMC6444527; doi:10.1186/s12910-019-0356-z)
Supplement: Supplementary file 2 — Invitation to Research. The text of the first email contact with survey participants inviting them to participate in the study. (DOCX 121 kb) [file 12910_2019_356_MOESM2_ESM.docx]

**Invitation to Research**

The following text was the first email distributed to survey participants

Subject line:

**Invitation to survey of Canadian medical students on physician-assisted dying**

Email text:

Dear Medical Student,

You are invited to participate in a nationwide study of Canadian medical students about your **views and perceptions on physician-assisted dying**. As future medical practitioners, your opinions on this issue are important. Your status as a current medical student in a Canadian university has made you eligible for this survey. All medical students in Canada will receive this survey, available in English or French. Medical students with any range of opinions (or no opinion) about physician-assisted dying are encouraged to participate. Because this survey is privately distributed to medical students only, it is important that you **do not share** or forward this survey invitation, or the web link for the survey, to anyone.

Click here to read the consent document and begin the survey: [https://surveys.mcgill.ca/limesurvey/[survey ID redacted]](https://surveys.mcgill.ca/limesurvey/index.php?sid=72599)

This survey has received ethical approval, is completely anonymous, and does not collect any personally-identifying information or metadata. Results will never be published in a way that could identify respondents. It should take you 15-20 minutes to complete. Following the survey, you will be redirected to an external webpage where you can choose to enter a draw to win one of 5 cash prizes of $100. Both the survey and prize draw are completely voluntary. Your information collected for the the prize draw cannot be linked to your survey responses in any way.

Thank you for your participation in this survey.  Your participation will help us develop a more complete evidence-based understanding of the future of physician-assisted dying in Canada.

The MedPASS team is comprised of researchers from the Faculty of Medicine, Faculty of Law, and Department of Sociology at McGill University and the University of Alberta. We are a non-partisan group interested in understanding the medical, legal, and ethical dimensions of assisted dying in the context of Canada’s changing legal landscape. Any questions or concerns can be directed to the research team at: [mcgillmedpass@gmail.com](mailto:mcgillmedpass@gmail.com)
