# Supplementary material for: Perceptions and intentions toward medical assistance in dying among Canadian medical students
Source: BMC Med Ethics. 2019 Apr 2;20:22. doi: 10.1186/s12910-019-0356-z (PMC6444527; doi:10.1186/s12910-019-0356-z)
Supplement: Supplementary file 3 — Informed Consent document. Copy of the informed consent document that preceded the online survey questionnaire. (DOCX 142 kb) [file 12910_2019_356_MOESM3_ESM.docx]

**Informed consent document**

Perceptions of physician-assisted dying among Canadian medical students: implications for policy and practice

You are invited to participate in a study of Canadian medical students, to survey your views and perceptions on physician-assisted dying. As future medical practitioners, your opinions on this issue are extremely valuable. Students with any range of opinions (or even no opinion) on physician-assisted dying are encouraged to contribute to this study. Your status as a current medical student in a Canadian university has made you eligible for this study. All current medical students in Canada will receive an invitation to this survey. Participation is voluntary.

**Purpose**: No empirical data currently exist on the perceptions of medical students about the issue of physician-assisted dying (also called medical assistance in dying) in Canada. An evidence basis is needed for the creation of policy, legal frameworks, professional guidelines, and medical education. Your participation will significantly contribute to the success of this study.

**Requirements**: Participation in this study requires you to complete a 15-minute anonymous survey. You will be asked questions about your views, opinions, perceptions, and experiences about physician-assisted dying, as well as some questions about your background, future ambitions, and medical education. Please pay close attention to the questions and response options, and answer honestly.

**Privacy**: This survey is completely anonymous and does not collect any personally-identifying information or metadata. Results will not be published in a way that could identify respondents.

**Risks**: Due to the subject matter of the survey, there is a risk of psychological or emotional discomfort. You may refuse to answer any question or withdraw from the study at any time. Because the survey is anonymous, we cannot identify your responses to withdraw you from the study after you have completed and submitted your survey). If you experience distress at any time during the completion of this survey and require assistance, please contact the counselling and mental health service at your university at **this link**.

There is a risk of personal, social, professional, or legal consequences if you disclose any illegal or unethical activity during the survey. We do not ask about illegal or unethical activity, and you should not disclose it during the survey. We guarantee anonymity for your survey responses to protect against these risks. We have designed the data collection such that no researcher can identify you, even if we wanted to. You should complete the survey privately and keep your responses private. Survey data will be stored for an indefinite period on an encrypted, password-protected hard drive and made accessible exclusively to analysts on the project team.

**Benefits**: There is no personal benefit to participating in this study. However, this study has benefits for the profession of medicine and medical education. No Canadian data currently exist on the perspective of medical students on the issue of physician-assisted dying. This research will provide important data on how future physicians perceive this practice on an ethical, social, and professional level. Survey results will be useful for drawing evidence-based conclusions on the future of physician-assisted dying in Canada, and for recommendations on regulatory frameworks, professional guidelines, and medical education. We will publish the results of this research through reports, academic presentations, policy papers, and journal articles.

**Compensation**: There is no compensation for participating in this study. However, following the survey, you will be redirected to an external webpage where you can choose to enter a draw to win one of 5 cash prizes of $100. Entering the draw is voluntary. Your name and email collected for the purposes of the prize draw cannot be linked to your survey responses in any way, will only be accessible to one independent analyst on the research team (Zachary Shefman), and will be securely deleted following the draw.

**Conflict of interest**: The researchers have no personal, professional, or financial stake in the results of this survey or the legal status of medical assistance in dying. We are an independent and non-partisan research team, not sponsored by any public or private stakeholders, institutions, or corporations, and with no conflicts of interest.

Thank you.

This study has been reviewed and given ethics approval by the *McGill Research Ethics Board* and the *Newfoundland and Labrador Health Research Ethics Board*.  If you have any questions about taking part in this study, you can contact the principal investigator, James Falconer, at [mcgillmedpass@gmail.com](mailto:mcgillmedpass@gmail.com). Or you can talk to someone who is not involved with the study, but who can advise you on your rights as a participant in a research study, by contacting the McGill Ethics Manager at 514-398-6831, or lynda.mcneil@mcgill.ca. In Newfoundland and Labrador, you can contact the Ethics Office at 709-777-6974, or email info@hrea.ca. [Click here to download or print a copy of this consent form for your records]

Researchers:

Dr. James Falconer (Principal investigator), Dept. of Sociology, University of Alberta

Félix Couture, MDCM candidate, Faculty of Medicine, McGill

Koray Demir, MDCM candidate, Faculty of Medicine, McGill

Michael Lang, BCL/LLB candidate, Faculty of Law, McGill

Zachary Shefman, BCL/LLB candidate, Faculty of Law, McGill

Mark Woo, MDCM candidate, Faculty of Medicine, McGill

[mcgillmedpass@gmail.com](mailto:mcgillmedpass@gmail.com)

Supervisor: Dr. Jennifer Fishman, McGill University

Montreal, Quebec, Canada

jennifer.fishman@mcgill.ca

514-398-7403
